# Editorial: The rapidly changing face of pediatric diabetes during the obesity epidemic

**DOI:** 10.3389/fendo.2025.1615551

**Published:** 2025-06-20

**Authors:** Dalia Al-Abdulrazzaq, Pratik Shah, Ibrahim Al-Alwan

**Affiliations:** ^1^ Faculty of Medicine, Kuwait University, Kuwait City, Kuwait; ^2^ Barts Health National Health Service (NHS) Trust, London, United Kingdom; ^3^ Department of Pediatrics, King Saud bin Abdulaziz University for Health Sciences Riyadh, Riyadh, Saudi Arabia

**Keywords:** diabetes mellitus, obesity, type 1 diabetes, type 2 diabetes, epidemic

Over the past two decades, the global pediatric landscape has undergone a profound shift driven by the rapid rise in childhood obesity. This trend, once predominantly a concern of high-income nations, now spans continents, socioeconomic groups, and ethnicities, altering the clinical profile and epidemiological patterns of pediatric diabetes. Once typified as two distinct diseases—Type 1 diabetes (T1D), as autoimmune and Type 2 diabetes (T2D) as metabolic—the current clinical realities reflect a more nuanced spectrum, frequently complicated by the coexistence of obesity, insulin resistance, and metabolic dysfunction in both forms.

The Research Topic “*The rapidly changing face of pediatric diabetes during the obesity epidemic”* brings together a collection of six studies that illuminate this evolving paradigm. These contributions explore diverse yet interlinked dimensions of pediatric diabetes, including genetic susceptibilities, epidemiological trends, treatment efficacy, disease course, and remission predictors—all within the context of rising obesity rates.

## Aims and objectives of the Research Topic

The aim of this Research Topic is to synthesize emerging evidence that characterizes how obesity is altering the pathophysiology, presentation, and progression of diabetes in children and adolescents. By examining novel treatment combinations, population-level trends, metabolic predictors, and genetic risk factors, these studies collectively aim to clarify how pediatric diabetes is changing, and what strategies may help clinicians, researchers, and public health officials respond effectively.

Our objective is twofold: first, to deepen the understanding of how obesity intersects with both T1D and T2D in youth, and second, to identify actionable pathways for improved diagnosis, management, and prevention in this high-risk population.

## Summary of contributing articles

The first study, *The Metabolic Effect of Combined Liraglutide Treatment and Lifestyle Modification on Obese Adolescents in a Tertiary Center in Riyadh*, provides important real-world data on the efficacy of GLP-1 receptor agonist therapy in pediatric populations. By combining pharmacologic intervention with structured lifestyle modification, this study demonstrates significant improvements in metabolic parameters including weight, glycemic control, and insulin sensitivity in obese adolescents. These findings underscore the potential of early combination therapies to disrupt the progression toward overt diabetes and metabolic syndrome in high-risk youth.

In *BMI Trajectories Among Children Diagnosed with Type 1 Diabetes Mellitus at a Tertiary Diabetes Center*, researchers investigate the longitudinal weight patterns in children with T1D. Their findings reveal a concerning trend: a significant proportion of children with T1D now present with or develop overweight and obesity during the course of their disease. This has implications for insulin dosing, glycemic variability, and the risk of long-term complications. The study challenges traditional assumptions that T1D is exclusively a lean phenotype and highlights the need for integrated weight management in this group.

The third article, *Comparison of Incidence Trends of Early-Onset and Late-Onset Type 2 Diabetes in the Asia-Pacific Region: A Joint Point Regression Analysis Based on the Global Burden of Disease Study 2022*, broadens the view with a regional epidemiologic analysis. By leveraging large-scale data, the study shows a steep rise in the incidence of early-onset T2D in several Asia-Pacific countries. This reflects not only a demographic and nutritional transition in these societies but also signals an urgent need for region-specific prevention and policy responses.

The article *Body Mass Index and Partial Remission in 119 Children with Type 1 Diabetes: A Six-Year Observational Study* addresses the controversial relationship between obesity and the “honeymoon phase” of T1D. Contrary to some expectations, this study finds that higher BMI is not associated with a higher likelihood of partial remission, suggesting that obesity-related insulin resistance may actually suppress residual β-cell function in early disease. This has important clinical implications for predicting disease progression and tailoring early management strategies.

The fifth contribution, *The rs17782313 polymorphism near MC4R gene confers a high risk of obesity and hyperglycemia, while PGC1α rs8192678 polymorphism is weakly correlated with glucometabolic disorder: a systematic review and meta-analysis*, brings genetic insight into the picture. Through a systematic review and meta-analysis, the authors confirm that MC4R polymorphisms significantly increase the risk of obesity and hyperglycemia in pediatric populations. Meanwhile, the contribution of PGC1α variants appears weaker and more context-dependent. These findings reinforce the relevance of genetic screening and precision medicine approaches in the early identification of children at risk.

Lastly, *A Single Point in Insulin Sensitivity Estimator of 5.4 is a Good Predictor of Both Metabolic Syndrome and Insulin-Resistant Adolescence with Obesity* introduces a practical tool for early identification of insulin resistance and metabolic syndrome. The proposed estimator offers a non-invasive, clinically feasible method to assess metabolic risk, potentially guiding early interventions before the onset of overt diabetes. This kind of tool is particularly timely given the increasing overlap of obesity, insulin resistance, and dysglycemia in pediatric populations.

## Broader context and implications

Together, these articles highlight a central truth: pediatric diabetes is no longer defined solely by autoimmunity or genetic predisposition. Rather, it increasingly exists at the intersection of metabolic, environmental, and behavioral factors—many of which are modifiable. The growing prevalence of obesity not only accelerates the onset of T2D in youth but also alters the course and clinical expression of T1D. It is also reshaping therapeutic responses, diminishing the likelihood of remission, and complicating long-term management.

These studies contribute to a reframing of pediatric diabetes as a multifaceted and dynamic condition. They collectively emphasize the need for a life-course approach to metabolic health that begins early in childhood. Interventions must integrate pharmacology, nutrition, behavioral support, and, increasingly, genomics to meet the changing needs of today’s youth.

In conclusion, the contributions in this Research Topic signal both a challenge and an opportunity. While the obesity epidemic poses a serious threat to pediatric metabolic health, it also provides a chance to reimagine diabetes prevention and care. By embracing interdisciplinary, culturally sensitive, and data-driven approaches, we can better support children living with or at risk for diabetes in this rapidly changing phase.

